# Effect of climate change-induced water-deficit stress on long-term rice yield

**DOI:** 10.1371/journal.pone.0284290

**Published:** 2023-04-17

**Authors:** Hungyen Chen, Yi-Chien Wu, Chia-Chi Cheng, Chih-Yung Teng

**Affiliations:** 1 Department of Agronomy, National Taiwan University, Taipei, Taiwan; 2 Taichung District Agricultural Research and Extension Station, Council of Agriculture, Changhua, Taiwan; CRIDA: Central Research Institute for Dryland Agriculture, INDIA

## Abstract

The water requirements of crops should be investigated to improve the efficiency of water use in irrigated agriculture. The main objective of the study was to assess the effects of water deficit stress on rice yields throughout the major cropping seasons. We analyzed rice yield data from field experiments in Taiwan over the period 1925–2019 to evaluate the effects of water-deficit stress on the yield of 12 rice cultivars. Weather data, including air temperatures, humidity, wind speed, sunshine duration, and rainfall were used to compute the temporal trends of reference evapotranspiration and crop water status (CWS) during rice growth stages. A negative CWS value indicates that the crop is water deficient, and a smaller value represents a lower water level (greater water-deficit stress) in crop growth. The CWS on rice growth under the initial, crop development, reproductive, and maturity stages declined by 96.9, 58.9, 24.7, and 198.6 mm in the cool cropping season and declined by 63.7, 18.1, 8.6, and 3.8 mm in the warm cropping season during the 95 years. The decreasing trends in the CWSs were used to represent the increases in water-deficit stress. The total yield change related to water-deficit stress on the cultivars from 1925–1944, 1945–1983, and 1996–2019 under the initial, crop development, reproductive, and maturity stages are -56.1 to 37.0, -77.5 to -12.3, 11.2 to 19.8, and -146.4 to 39.1 kg ha^-1^ in the cool cropping season and -16.5 to 8.2, -12.9 to 8.1, -2.3 to 9.0, and -9.3 to 8.0 in the warm cropping season, respectively. Our results suggest that CWS may be a determining factor for rice to thrive during the developmental stage, but not the reproductive stage. In addition, the effect of water-deficit stress has increasingly affected the growth of rice in recent years.

## Introduction

Global climate change, including increased temperatures and fluctuating rainfall, has become a threat with a high potential to affect the water supply and agricultural sectors [1–3]. The increase in temperature and decrease in rainfall negatively affects the growth of plants because plants are subjected to temperature and water stress due to an increase in evapotranspiration [4, 5]. The impact of climate change on the hydrological cycle, water balance, and runoff characteristics has emerged as a significant stressor at local and district levels, although there are uncertainties regarding the impacts of climate variability on water resources [6, 7]. It is suggested that water availability and crop productivity will decrease significantly, and climate change will have an impact on irrigation water requirements and crop yield [8, 9]. Crop yield change is expected due to the shifting growth phase and photosynthetic capacity, and increasing respiration and water requirements, which result from climate change [10, 11]. To investigate the general effects of crop yield change on climate change, it is necessary to analyze long-term temporal variations between crop yields and climate variables [12–14].

To improve water-use efficiency in irrigated agriculture, it is important to study and understand crop water requirements. Evapotranspiration is a vital component when describing the hydrological cycle in ecological systems, estimating water balance, and determining water availability along with precipitation [15, 16]. Reference evapotranspiration (ET_0_) is a parameter of climatic conditions that has been widely investigated as an indicator of climate change [17, 18]. Crop evapotranspiration (ET_C_) is a variable for the optimization of irrigation water productivity and designing the schedule of irrigation in the implementation of agricultural water management [19] (Gong et al., 2020) and is highly influenced by irrigation water supply under different irrigation levels [20, 21]. The Penman-Monteith (PM) model based on the Food and Agriculture Organization (FAO)-56 guidelines has served as a reference method because it produces the most accurate results compared to lysimetric measurements [22, 23]. The PM model has been widely used for the estimation of daily or monthly ET_0_ in different agro-climatic zones by many researchers for decades [22, 24, 25].

Rice is a semi-aquatic plant that depends on the rainfall and temperature of the cultivation area and hence, is heavily affected by climate change [5, 26]. Severe effects of drought and high temperature on the growth and yield of rice due to insufficient water supply and improper scheduling of irrigation have been reported [27, 28]. Some reports have revealed that rice yield may be affected by temperature and precipitation because of some physiological mechanisms [29, 30]. Although many studies have revealed the impacts of climate change on crop production utilizing climate model projections of temperatures and rainfall [31, 32], the number of studies that analyze the effect of water-deficit stress on rice yield using long-term field experimental data is limited.

Water deficit stress occurs when the amount of water required is greater than the amount of water available during a certain time. Our goal was to assess the effects of water deficit stress on rice yields throughout the major cropping seasons. In this study, we analyzed the yield data of 12 rice cultivars in cool and warm cropping seasons, separately, from field experiments conducted under irrigated conditions with optimal management at a research station in Taichung, Taiwan over the period 1925–2019. First, weather data, including average, maximum, and minimum temperatures, humidity, wind speed, and sunshine duration, collected at the research farm were used to compute the long-term temporal trends of reference evapotranspiration during the initial, crop development, reproductive, and maturity stages of rice growth during the 95 years. Second, the crop evapotranspiration of rice under the growth stages was calculated using the estimated reference evapotranspiration and crop coefficient of rice. Third, the crop water status during the growth stages was calculated using the estimated crop evapotranspiration and collected rainfall data. Fourth, long-term temporal trends in crop water status during the growth stages were deduced to reveal the temporal trend in water-deficit stress. Fifth, a multiple linear regression model was applied to evaluate the relationships between rice grain yield and water-deficit stress during the four growth stages. Finally, total yield changes computed from the regression coefficients for each growth stage over the periods 1925–1944, 1945–1983, and 1996–2019 were used separately to reveal the effects of water-deficit stress on rice yield and the temporal variations during the experimental period.

## Materials and methods

### Field experiment

A field experiment on rice growth in two cropping seasons was conducted from 1925 to 2019 at the Taichung District Agricultural Research and Extension Station, Council of Agriculture, Executive Yuan, Taiwan (1925–1983: 24º09′ N 120º41′ E, altitude 77 m above mean sea level; 1996–2019: 24º00′ N 120º32′ E, altitude 19 m above mean sea level). The rice seeds were sown in the cool cropping season in mid-January, and the seeded area was dibbled either in February or March every year over the period 1925–2019 except for 1948–1951, 1985–1995, and 2014–2016. Rice from the cool cropping season was harvested either in June or July. The rice seeds were sown in the warm cropping season in June, and the area was dibbled either in July or August every year over the period 1925–2019, except for 1945, 1947–1951, 1985–1995, and 2013–2015. Rice in the warm cropping season was harvested either in October or November. The seedlings were transplanted into the fields by hand. The area of the plot for each cultivar was 27 m^2^. Continuous flooding irrigation to 5 cm above the soil surface was carried out in the field during the period between transplanting and drying. Re-irrigation was applied when the field water subsided to the soil surface. The grain yield was obtained by harvesting from all the hills in the plots (at a grain maturity rate of 98%), except for the side rows, and then measuring the grain weight. No field permits were required for this work at the research station which the authors are affiliated with.

### Rice yield data

Twelve rice cultivars were used throughout the experimental period in cool and warm cropping seasons, separately. In cool cropping season, Nakamura (NM; 1925–1931), Taichung S2 (TCS2; 1925–1932), Baiker (BK; 1925–1944), Taichung S6 (TCS6; 1933–1944), Wugen (WG; 1925–1947, 1952–1976), Baimifun (BMF; 1945–1947, 1952–1976), Taichung 65 (TC65; 1930–1947, 1952–1983), Taichung 150 (TC150; 1945–1947, 1952–1983), Taiagro 67 (TA67; 1996–2013, 2017–2019), Taichung 189 (TC189; 1996–2013, 2017–2019), Taichung Indica 10 (TCI10; 1996–2013, 2017–2019), and Tai Japonica 9 (TJ9; 2000–2013, 2017–2019) were used. In warm cropping season, Nakamura (NM; 1925–1931), Taichung S2 (TCS2; 1925–1944), Jingou (JG; 1925–1944), Nyaoyao (NY; 1925–1944), Swanjian (SJ; 1946, 1952–1976), Sianlou (SL; 1946, 1952–1976), Taichung 65 (TC65; 1930–1944, 1946, 1952–1983), Taichung 150 (TC150; 1946, 1952–1983), Taiagro 67 (TA67; 1996–2012, 2016–2019), Taichung 189 (TC189; 1996–2012, 2016–2019), Taichung Indica 10 (TCI10; 1996–2012, 2016–2019), and Tai Japonica 9 (TJ9, 2000–2012, 2016–2019) were used.

For each cropping season, three groups of cultivars with overlapping cultivation periods were clustered together to calculate the group average value, which represents the effect of water stress on rice yield in each period. Based on the cultivation period among cultivars, four cultivars were included in the 1925–1944, 1945–1983, and 1996–2019 periods, separately.

Four distinct stages of rice growth were used for the analyses. For the cool cropping season, the initial stage was from March 1–31, the crop development stage was from April 1–30, the reproductive (mid-season) was from May 1–31, and the maturity (late season) stage was from June 1–30. For the warm cropping season, the initial stage was from August 1–31, the crop development stage was from September 1–30, the reproductive (mid-season) stage was from October 1–31, and the maturity (late season) stage was from November 1–30.

### Weather data

A weather station was set up on the research station farm. The site is surrounded by field crops and the topography is flat. Daily weather data recording began on January 1, 1925. Meteorological instruments at the station included a solarimeter, glass thermometers for minimum and maximum temperatures, a psychrometer, and a thermo-hygrograph. The air temperature, humidity, wind speed, rainfall, and sunshine duration during the cropping seasons throughout the experimental period were used for the analyses. The average, minimum, and maximum temperatures, average relative humidity, and average wind speed (2 m above the soil surface) under the four growth stages for each year were calculated as the average of the daily values in the cool and warm cropping seasons, respectively. Rainfall and sunshine durations under the four growth stages for each year were calculated as the sum of the daily values in the two cropping seasons.

### Statistical models

The reference evapotranspiration was determined using the Penman–Monteith model [22], which was suggested by the Food and Agriculture Organization of the United Nations (FAO) and the International Commission for Irrigation and Drainage as a universal model for calculating reference evapotranspiration:

$${\text{ET}}_{0}=\frac{0.408\Delta \left({\text{R}}_{\text{n}}-\text{G}\right)+\gamma \frac{900}{\text{T}+273}{\text{u}}_{2}\left({\text{e}}_{\text{s}}-{\text{e}}_{\text{a}}\right)}{\Delta +\gamma \left(1+0.34{\text{u}}_{2}\right)}$$ (1)

where ET_0_ is the reference evapotranspiration (mm day^-1^), R_n_ is the net radiation at the crop surface (MJ m^-2^ day^-1^), G is the soil heat flux density (MJ m^-2^ day^-1^), γ is the psychrometric constant (kPa °C^-1^), T is the average air temperature (°C), u_2_ is the wind speed at a height of 2 m (m s^-1^), e_s_ is the saturation vapor pressure (kPa), e_a_ is the actual vapor pressure (kPa), and Δ is the slope vapor pressure curve (kPa °C^-1^). The detailed calculation of the parameters is described elsewhere [22, 33]. The ET_0_ calculation was conducted using the ET_0_ calculator [34] according to the calculation procedures outlined in the FAO irrigation and drainage paper 56 [22].

The crop evapotranspiration (ET_C_) of the rice during the growth stages was calculated under standard conditions. It was assumed that crop growth or evapotranspiration were not limited by soil water and salinity stress, crop density, pests and diseases, weed infestation, or low fertility. ET_C_ (mm day^-1^) was determined by the crop coefficient approach [22], whereby the effects of the weather conditions are incorporated into ET_0_ (mm day^-1^) and the crop characteristics into the crop coefficient (K_C_):

$${\text{ET}}_{\text{C}}={\text{ET}}_{0}\times {\text{K}}_{\text{C}}$$ (2)

The Kc values for rice during the initial, crop development, reproductive (mid-season), and maturity (late season) stages were 1.15, 1.23, 1.14, and 1.02, respectively, as estimated by Tyagi et al. [35].

The effective rainfall was calculated using the formula described in the FAO irrigation water management paper [36]:

$$\text{PE}=\left\{\begin{array}{c} 0.8\text{P}\;–\;25,\;\;\text{if}\;\text{P}\;\geq \;75\;\text{mm}\;{\text{month}}^{-1}\\ 0.6\text{P}\;–\;10,\;\;\text{if}\;\text{P}\;\leq \;75\;\text{mm}\;{\text{month}}^{-1} \end{array}\right.$$ (3)

where PE is effective rainfall (mm month^-1^), and P is rainfall (mm month^-1^).

Crop water status (CWS) of rice in the growth stage was calculated as:

$$\text{CWS}=\text{PE}–{\text{ET}}_{\text{C}}–\text{PERC}–\text{WL}$$ (4)

where PE is the effective rainfall (mm month^-1^), ET_C_ is the crop evapotranspiration of rice (mm day^-1^), PERC is the amount of percolation and seepage losses (mm day^-1^), and WL is the amount of water required to establish a water layer (mm month^-1^). For analysis, the period of each growth stage was suggested as being consistent with the period of one single month during the long-term experimental period. To determine the total ET_C_ and PERC values throughout the entire growth stage, their daily values were multiplied by the number of days in each growth stage. The PERC was suggested to be 5 mm day^-1^ in the analysis based on well-saturated clay soils at the research farm [36]. The WL was assumed to be 100 mm throughout the growth stage [36].

The value of the CWS represents the water status of crop growth under weather conditions. A negative CWS value indicates that the crop is water deficient, and a smaller value represents a lower water level (greater water-deficit stress) in crop growth. In cases where all the water needed for optimal crop growth is provided by rainfall, irrigation is not required, and the CWS equals zero. The CWS determined in this study was inspired by the formula for irrigation water need (IN) [36], IN = ET_C_ + PERC + WL—PE. The value of the CWS equals the negative number of the value of IN.

A simple linear regression equation was used to represent the linear time trend of the CWS. The underlying relationship between *x* and *t* can be described by

$$\text{x}=\beta_{0}+\beta_{1}\text{t}+\varepsilon$$ (5)

where x is water stress, t is the year corresponding to x, *β*_0_ is the intercept, *β*_1_ is the regression coefficient representing the rate of change in the CWS per year, and *ε* is the model error.

A multiple linear regression model was used to describe the relationship between rice yield and water stress, using the value of rice yield as the response variable and the values of CWS during the initial, crop development, reproductive, and maturity stages (CWS_I_, CWS_D_, CWS_R_, and CWS_M_) as explanatory variables for each rice cultivar in each cropping season.

$$\text{Y}=\beta_{0}+\beta_{{\text{CWS}}_{\text{I}}}{\text{CWS}}_{\text{I}}+\beta_{{\text{CWS}}_{\text{D}}}{\text{CWS}}_{\text{D}}+\beta_{{\text{CWS}}_{\text{R}}}{\text{CWS}}_{\text{R}}+\beta_{{\text{CWS}}_{\text{M}}}{\text{CWS}}_{\text{M}}+\varepsilon$$ (6)

where *β*_0_ represents the model intercept, *β* is the regression coefficient for water stress in each growth stage, and *ε* is the model error.

The total rice yield change (kg ha^-1^) related to water stress under each growth stage was computed using the regression coefficients for water stress (*β*_CWS_) and the estimated change in crop water status (ΔCWS) throughout each cultivation period.


$$\text{Total}\;\text{yield}\;\text{change}=\beta_{\text{CWS}}\times {\Delta \text{CWS}}_{\text{Total}\;\text{years}}$$ (7)

## Results

### Long-term temporal variations in rice yield

**Fig 1:**
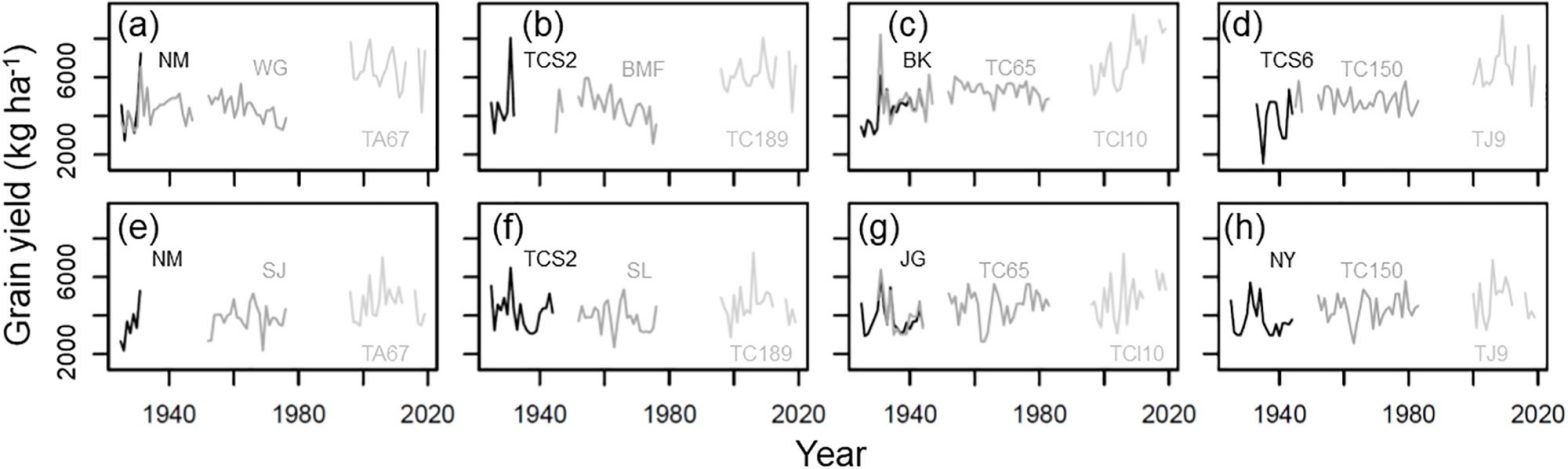
Yearly variation in grain yield and cultivation period for twelve rice cultivars in cool (a, b, c, and d) and warm (e, f, g, and h) cropping seasons.

In the cool cropping season, the yield of the four rice cultivars for the period 1925–1944 ranged between 1,530 and 8,055 kg ha^-1^, with an average ± standard deviation (SD) of 4,236 ± 1,114 kg ha^-1^. The yield of the four rice cultivars for the period 1945–1983 ranged between 2,550 and 8,215 kg ha^-1^, with an average ± SD of 4,708 ± 774 kg ha^-1^. The yield of the four rice cultivars during 1996–2019 ranged between 4,181 and 9,268 kg ha^-1^, with an average ± SD of 6,523 ± 1,080 kg ha^-1^ (Fig 1a–1d). In the warm cropping season, the yield of the four rice cultivars for the period 1925–1944 ranged between 2,181 and 6,493 kg ha^-1^, with an average ± standard deviation (SD) of 3,880 ± 873 kg ha^-1^, the yield of the four rice cultivars for the period 1945–1983 ranged between 2,180 and 6,384 kg ha^-1^, with an average ± SD of 4,149 ± 796 kg ha^-1^, and the yield of the four rice cultivars for the period 1996–2019 ranged between 2,861 and 7,269 kg ha^-1^, with an average ± SD of 4,704 ± 971 kg ha^-1^ (Fig 1e–1h).

### Long-term temporal variations in reference evapotranspiration

**Fig 2:**
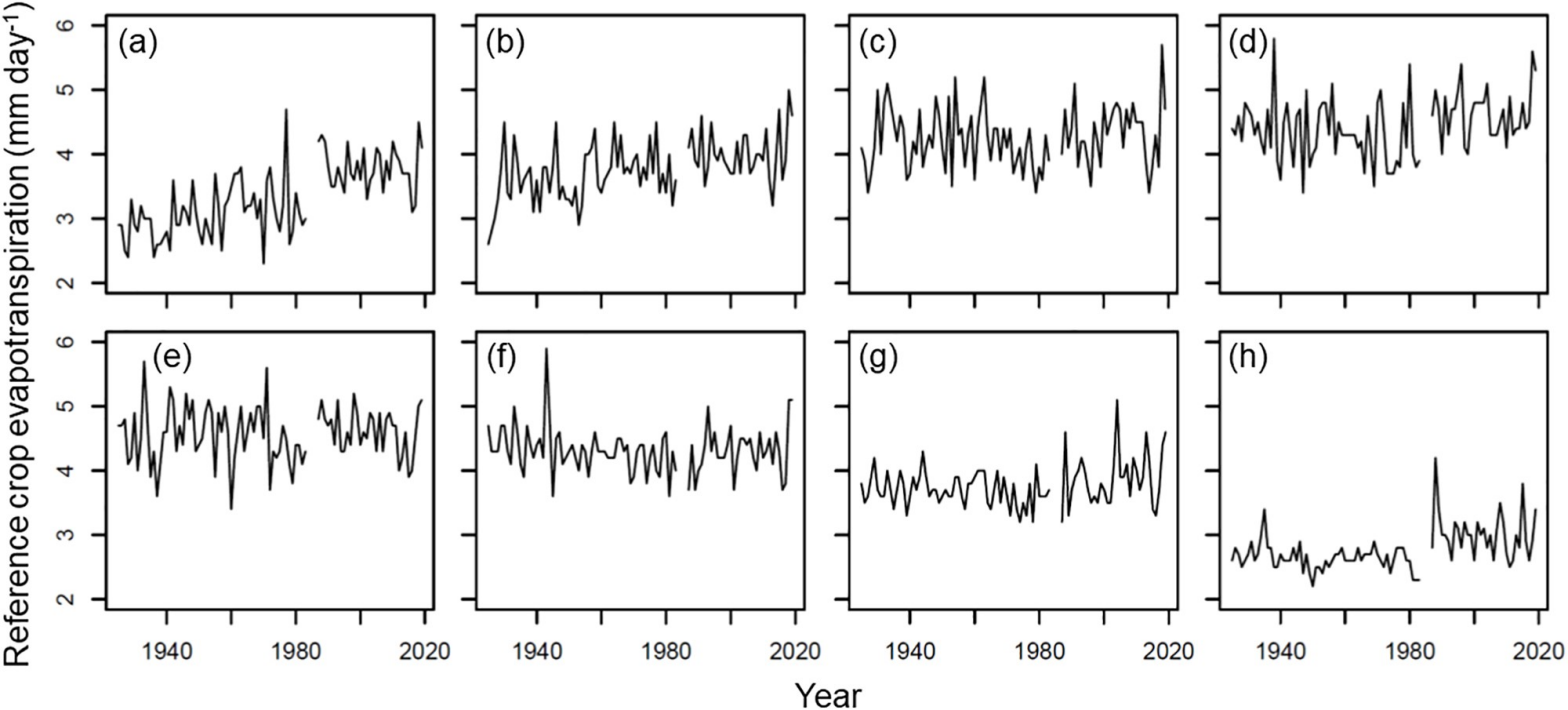
Yearly variation in reference evapotranspiration under initial (a and e), crop development (b and f), reproductive (c and g), and maturity (d and h) stages of rice in cool (a, b, c, and d) and warm (e, f, g, and h) cropping seasons.

In the cool cropping season, the ET_0_ ranged between 2.3 to 4.7, 2.6 to 5.0, 3.4 to 5.7, and 3.4 to 5.8 mm day^-1^ under the initial, crop development, reproductive, and maturity stages, respectively (Fig 2a–2d). The average values ± SDs of ET_0_ were 3.3 ± 0.5, 3.8 ± 0.4, 4.3 ± 0.5, and 4.4 ± 0.5 mm day^-1^ under the four growth stages, respectively (Fig 2a–2d). In the warm cropping season, the ET_0_ ranged between 3.4–5.7, 3.6–5.9, 3.2–5.1, and 2.2–4.2 mm day^-1^ under the initial, crop development, reproductive, and maturity stages, respectively (Fig 2e–2h). The average values ± SDs of ET_0_ were 4.6 ± 0.4, 4.3 ± 0.4, 3.8 ± 0.3, and 2.8 ± 0.3 mm day^-1^ under the four growth stages, respectively (Fig 2e–2h).

### Long-term temporal variations in crop water status

**Fig 3:**
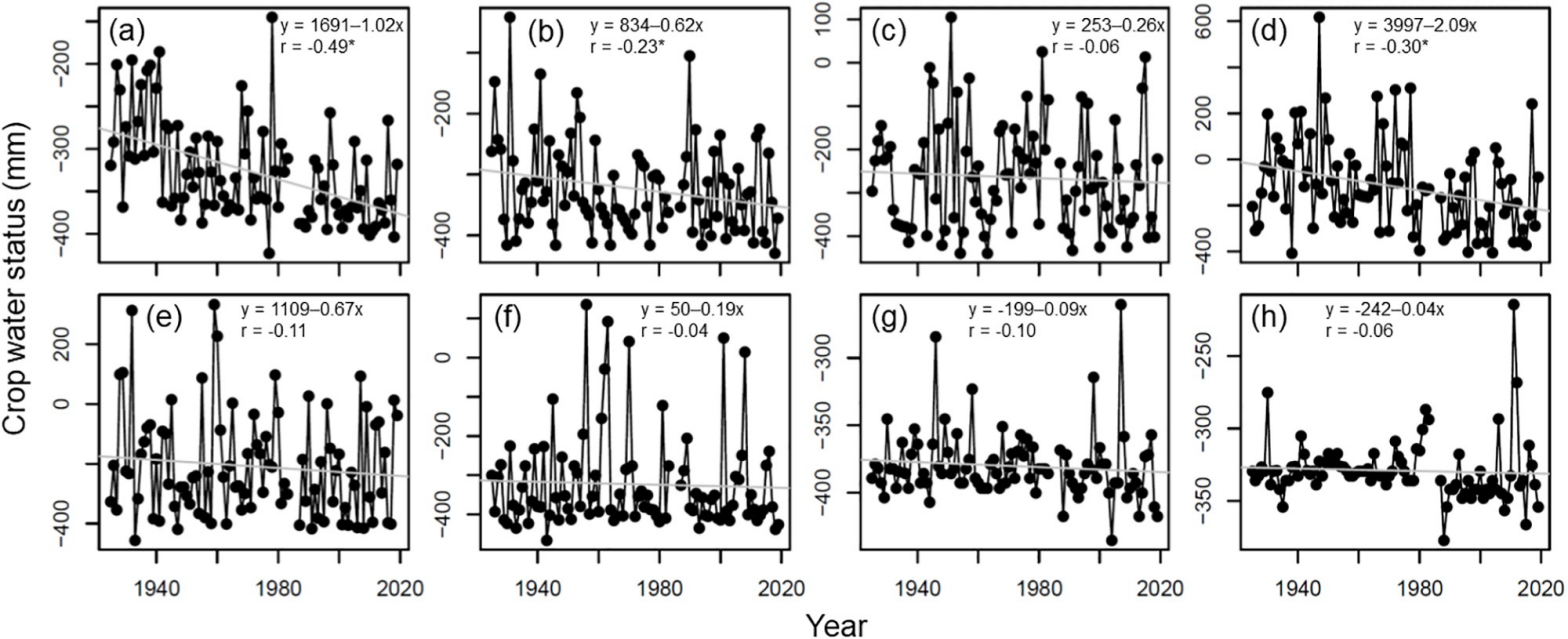
Yearly trend of crop water status on rice growth under initial (a and e), crop development (b and f), reproductive (c and g), and maturity (d and h) stages in cool (a, b, c, and d) and warm (e, f, g, and h) cropping seasons. Grey line represents the linear regression line. * represents p-value < 0.05.

In the cool cropping season, the CWS on rice growth under initial, crop development, reproductive, and maturity stages ranged between -422.4 to -145.0, -428.9 to -42.0, -438.8 to 105.0, and -407.0 to 617.3 mm, respectively (Fig 3a–3d). The average values ± SDs of CWS were -327.2 ± 58.6, -323.1 ± 75.4, -263.9 ± 122.7, and -177.9 ± 195.1 mm under the four growth stages, respectively (Fig 3a–3d). In the warm cropping season, the CWS on rice growth under initial, crop development, reproductive, and maturity stages ranged between -455.7 to 333.8, -466.2 to 136.4, -435.2 to -260.1, and -416.3 to -244.8 mm, respectively (Fig 3e–3h). The average values ± SDs of WDS were -208.3 ± 173.4, -322.9 ± 123.5, -380.2 ± 24.9, and -366.2 ± 24.3 mm under the four growth stages, respectively (Fig 3e–3h). The CWS on rice growth under the initial, crop development, reproductive, and maturity stages declined by 96.9, 58.9, 24.7, and 198.6 mm in the cool cropping season, respectively (Fig 3a–3d), and declined by 63.7, 18.1, 8.6, and 3.8 mm in the warm cropping season, respectively (Fig 3e–3h) from 1925 to 2019.

### Effects of crop water status on rice yield

**Fig 4:**
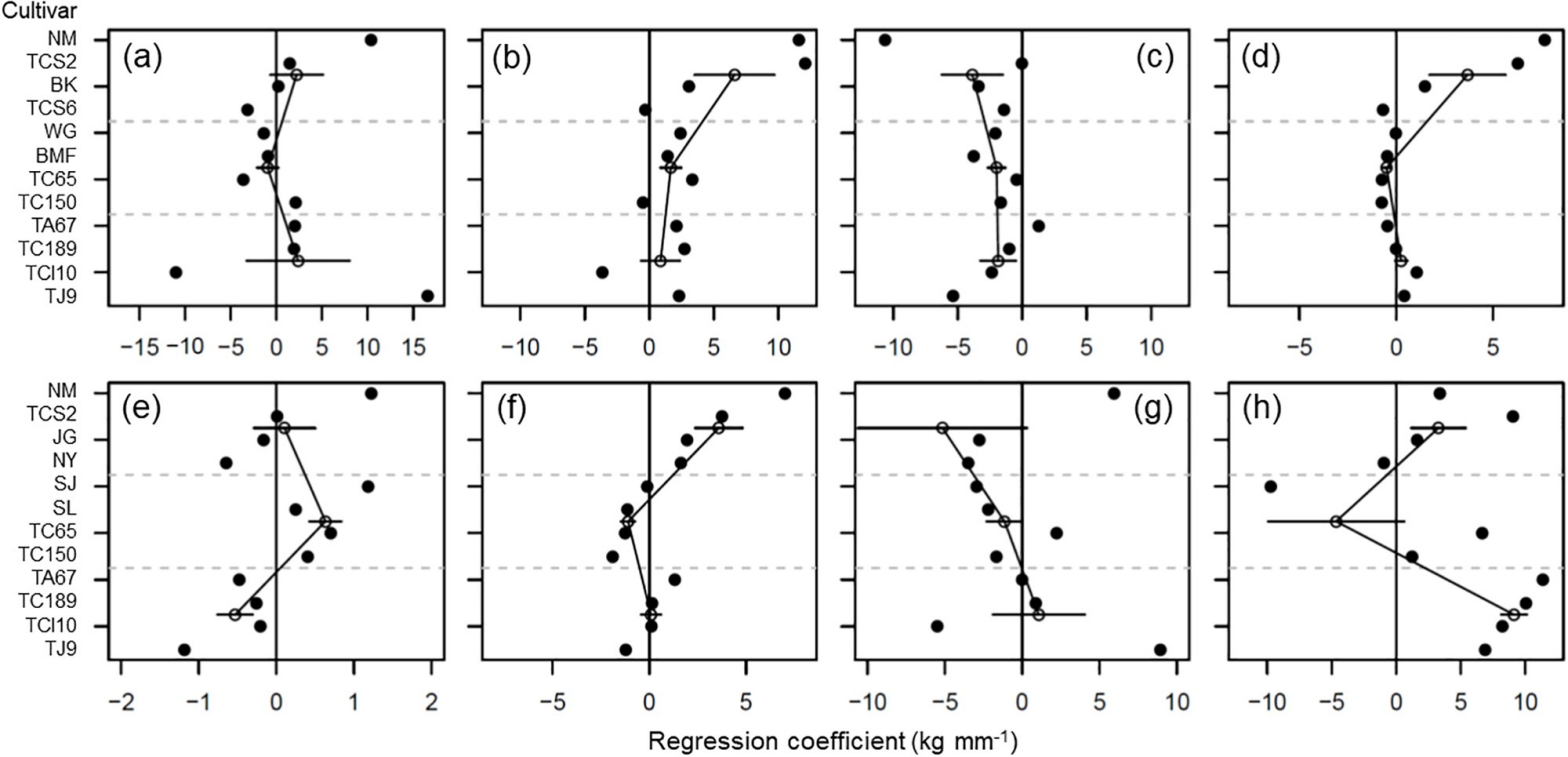
Regression coefficient of rice yield on crop water status under initial (a and e), crop development (b and f), reproductive (c and g), and maturity (d and h) stages in cool (a, b, c, and d) and warm (e, f, g, and h) cropping seasons. Filled circles represent the value of a cultivar. Open circles represent the average value of a group of cultivars having overlapped cultivation period. The length of the black line on the open circle represents the value of standard deviation. Horizontal dash lines separate the groups of cultivars having overlapped cultivation periods. In each panel, the upper, mid, and lower zone represent the periods of 1925–1944, 1945–1983, and 1996–2019, respectively.

The long-term temporal variation in the estimated effects of the CWS on the grain yield of 12 rice cultivars in the cool and warm cropping seasons from 1925 to 2019 are shown in Fig 4. A positive value of the regression coefficient reflects a coincident pattern between grain yield and CWS, and a negative value indicates an inverse response of grain yield to CWS. In the cool cropping season, the average regression coefficients under the initial and maturity stages revealed positive values in 1925–1944 and 1996–2019, but a negative average value over the period 1945–1983 (Fig 4a and 4d); the average regression coefficients under the crop development stage revealed all positive values and decreased throughout the experimental period (Fig 4b); and the average regression coefficients under the reproductive stage revealed all negative values and increased throughout the experimental period (Fig 4c). In the warm cropping season, the average regression coefficients under the initial stage revealed positive values in the periods 1925–1944 and 1945–1983, but a negative average value from 1996–2019 (Fig 4e). The average regression coefficients under the crop development and maturity stages revealed positive values in the periods 1925–1944 and 1996–2019, but a negative average value from 1945–1983 (Fig 4f and 4h). The average regression coefficients in the reproductive stage revealed a negative value from 1925–1944 and 1945–1983, but a positive value from 1996–2019, and increased throughout the experimental period (Fig 4g).

### Yield changes responding to crop water status

The mean total yield change relating to the CWS on the cultivars in cool cropping seasons over the periods 1925–1944, 1945–1983, and 1996–2019 are -43.2, 37.0, and -56.1 kg ha^-1^ in the initial stage, respectively, -77.5, -39.0, and -12.3 kg ha^-1^ in the crop development stage 19.3, 19.8, and 11.2 kg ha^-1^ in the reproductive stage, and -146.4, 39.1, and -12.4 kg ha^-1^ in the maturity stage, respectively (Table 1). The mean total yield change related to the CWS on the cultivars in the warm cropping seasons over the periods 1925–1944, 1945–1983, and 1996–2019 are -1.3, -16.5, and 8.2 kg ha^-1^ in the initial stage, -12.9, 8.1, and -0.4 kg ha^-1^ in the crop development stage, 9.0, 4.1, and -2.3 kg ha^-1^ in the reproductive stage, and -2.8, 8.0, and -9.3 kg ha^-1^ in the maturity stage, respectively (Table 1).

**Table 1:** Total rice yield change (kg ha^-1^) related to crop water status over the periods 1925–1944, 1945–1983, and 1996–2019 under initial (INI), crop development (DEV), reproductive (REP), and maturity (MAT) stages in the cool and warm cropping seasons.

		1925–1944				1945–1983				1996–2019			
		INI	DEV	REP	MAT	INI	DEV	REP	MAT	INI	DEV	REP	MAT
Cool cropping season	Mean	-43.2	-77.5	19.3	-146.4	37.0	-39.0	19.8	39.1	-56.1	-12.3	11.2	-12.4
	SD	112.3	72.7	23.5	156.2	92.0	38.5	13.7	27.0	265.2	42.9	16.7	31.0
Warm cropping season	Mean	-1.3	-12.9	9.0	-2.8	-16.5	8.1	4.1	8.0	8.2	-0.4	-2.3	-9.3
	SD	10.1	8.9	19.1	3.6	10.7	5.5	8.3	18.2	6.9	4.5	12.6	2.0

## Discussion

In most cultivated areas of Taiwan, two cropping seasons are maintained throughout the year. The cool cropping season starts in late February or early March (initial stage) and ends in late June (maturity stage), and the warm cropping season starts in late July or early August (initial stage) and ends in late November (maturity stage). The patterns of temperature variation are opposite in the two cropping seasons. The average temperature increases throughout the cool cropping season while the average temperature decreases throughout the warm cropping season. The patterns of variation in reference evapotranspiration under the four growth stages of rice were the opposite between the cool and warm cropping seasons (Fig 2). Thus, to reveal the climatic effect, the rice yield response to climate variables needs to be analyzed separately in the cool and warm cropping seasons. The value for Qiu et al. [37] differentiates the changes in the seasonal crop evapotranspiration of rice in terms of growth duration under varying types of warming patterns using an evapotranspiration estimation model. The reference evapotranspiration increased throughout the cool cropping season, whereas it decreased throughout the warm cropping season (Fig 2). The different patterns of temporal and spatial variation in the reference evapotranspiration and sensitivity coefficient responses to precipitation and temperature were investigated [38, 39].

Long-term decreasing trends and negative values in crop water status were observed at all four growth stages in the two cropping seasons (Fig 3). This result showed that increasing crop water deficiency led to greater water-deficit stress on rice growth. The decreased crop water status was due to the increased air temperature and decreased rainfall [1]. The water deficit is limiting the growth and productivity of crops and has been a major problem for crop production worldwide, especially in rain-fed agricultural areas [40–42]. In the cool cropping season, the decreasing trend of crop water status was severe during the initial and maturity stages and mild during the reproductive stage (Fig 3). Compared with the cool cropping season, the decreasing trend in crop water status in the warm cropping season was relatively small under the four growth stages (Fig 3). This result may be due to the greater temperature increase and rainfall decrease in the cool season as opposed to the warm season [43].

The rice yield changes related to the crop water status were negative during the rice development stage (except for the warm cropping season from 1945–1983). This result suggests that crop water may be a determining factor for rice growth during the development stage [22, 44]. The rice yield changes related to the crop water status were positive during the rice reproductive stage (except for the warm cropping season over the period 1996–2019). This result suggests that crop water may not be a determining factor for rice growth during the reproductive stage [22, 45]. In recent years, from 1996 to 2019, negative yield changes were observed under all four growth stages in the cool cropping season and under crop development, reproductive, and maturity stages in the warm cropping season. This result may suggest that water-deficit stress has had a greater effect on rice growth in recent years [46, 47].

The values of crop water status under the four growth stages had little correlation with each other in both the cool (| r | ≤ 0.24) and warm (| r | ≤ 0.16) cropping seasons (Table 2). The annual variations in the yields of the cultivars with overlapping cultivation periods were correlated with each other in the same groups (Fig 3). The correlation coefficients of the yearly yields among the rice cultivar pairs with overlapping cultivation periods averaged 0.838, 0.605, and 0.665 in the cool cropping season during 1925–1944, 1945–1983, 1996–2019, respectively, and 0.716, 0.566, and 0.735 in the warm cropping season during 1925–1944, 1945–1983, 1996–2019, respectively. The correlations between the changes in crop water status under the four growth stages may make it difficult to separate the effects of different growth stages due to the co-linearity [48]. Although these problems have been discussed, the observations at our station showed low to little correlation among the values at different growth stages during the cropping seasons.

**Table 2:** Correlation coefficients of crop water status among growth stages in the cool and warm cropping seasons.

		Cool cropping season					Warm cropping season		
	Growth stage	INI	DEV	REP		Growth stage	INI	DEV	REP
Cool cropping season	DEV	0.222*	-	-	Warm cropping season	DEV	-0.151	-	-
	REP	0.154	0.045	-		REP	-0.102	0.087	-
	MAT	0.235*	0.059	0.094		MAT	-0.074	-0.117	0.002

INI, DEV, REP, and MAT represent initial, crop development, reproductive, and maturity stages, respectively.

* represents p-value < 0.05.

The cultivars of japonica type rice in Taiwan are the only japonica type rice that can grow under relatively high temperatures and produce good-quality rice with a high yield [49, 50]. Crops in tropical regions have been reported to be more sensitive to warming because their temperature is already close to their optimum temperature during the growing period [3]. In many regions, a slight increase in temperature with sufficient rainfall may have a positive effect on crops [51]. The lowland rice varieties were reported to be highly sensitive to soil drying, and their yields decline when the soil dries below saturation [52].

Data were collected from the same research station during the long-term experimental period. To obtain general results, the analysis of the crop yield response to global or national water-deficit stress should be extended. Data collected in different areas or on different temporal and spatial scales may result in different conclusions [53, 54]. For example, up to 45% yield reductions of rice are expected by the end of this century due to climate change, including water deficit, in the countries in eastern Africa [55]. In Iran, it was reported that water deficit during vegetative, flowering and grain filling stages reduced mean grain yield by 21, 50 and 21% on average in comparison to control, respectively [56]. In this study, long-term temporal variation in the rice yield response to water-deficit stress was revealed, even though the rice cultivars varied throughout the study period. During an experimental period of over 90 years since 1925, it is impossible to maintain the crop yield experiments using the same cultivar and maintaining the same environmental and cultivational conditions consistently. It is also difficult to consider the factors that may affect the growth and production of crops, such as insects, diseases, and soil fertility [57–60], as well as human-induced effects, such as modern management, improving technology, and cultivator practices [26, 58] for long-term observations. Crop evapotranspiration could be influenced by other factors, such as soil condition, canopy cover, and the fraction of leaf senescence; thus, the information of these coefficients may be considered for the calculation of crop evapotranspiration, if possible [19, 22]. In addition, extreme climatic events, such as floods and heatwaves, may pose additional risks to crop production [61].

This study revealed the effect of water-deficit stress on rice yield in both cool and warm cropping seasons. The results provide long-term evidence of declining crop water status during the rice-growing seasons. The average values of ET_0_ were estimated as 3.3–4.4 mm day^-1^, and 2.8–4.6 mm day^-1^ in cool and warm cropping seasons, respectively, under the rice growth stages. The crop water status has decreased by 24.7–198.6 mm in the cool cropping season and 3.8–63.7 mm in the warm cropping season under the rice growth stages since 1925 and during the 95 years. Compared with the cool cropping season, the decreasing trend in crop water status in the warm cropping season was relatively slight under the four growth stages. The total water-deficit stress related yield change in the cultivars in the cool cropping season during 1925–1944, 1945–1983, and 1996–2019 were -56.1 to 37.0, -77.5 to -12.3, 11.2 to 19.8, and -146.4 to 39.1 kg ha^-1^ under the initial, crop development, reproductive, and maturity stages, respectively. The total yield change related to the CWS on the cultivars in the warm cropping season during 1925–1944, 1945–1983, and 1996–2019 are -16.5 to 8.2, -12.9 to 8.1, -2.3 to 9.0, and -9.3 to 8.0 kg ha^-1^ under the initial, crop development, reproductive, and maturity stages, respectively. Our results suggest that crop water may be a determining factor for rice growth during the developmental stage, but not during the reproductive stage. In addition, water-deficit stress has been increasingly affecting rice growth in recent years. To maintain high productivity and quality, our results on the effect of water-deficit stress on rice grain yield should be considered along with other adaptation strategies targeting agronomic efforts and breeding technologies.

## Supporting information

S1 File Field experimental data. (CSV)
